# Improving CNV Detection Performance Except for Software-Specific Problematic Regions

**DOI:** 10.3390/genes17010105

**Published:** 2026-01-19

**Authors:** Jinha Hwang, Jung Hye Byeon, Baik-Lin Eun, Myung-Hyun Nam, Yunjung Cho, Seung Gyu Yun

**Affiliations:** 1 Department of Laboratory Medicine, College of Medicine, Korea University, Seoul 02841, Republic of Korea; jinha1226@gmail.com (J.H.); yuret@korea.ac.kr (M.-H.N.); eqcho1ku@korea.ac.kr (Y.C.); 2 Department of Pediatrics, College of Medicine, Korea University, Seoul 02841, Republic of Korea; agnes4@korea.ac.kr (J.H.B.); bleun@korea.ac.kr (B.-L.E.)

**Keywords:** copy number variation, chromosomal microarray, whole exome sequencing

## Abstract

**Background/Objectives**: Whole exome sequencing (WES) is an effective method for detecting disease-causing variants. However, copy number variation (CNV) detection using WES data often has limited sensitivity and high false-positive rates. **Methods**: In this study, we constructed a reference CNV set using chromosomal microarray analysis (CMA) data from 44 of 180 individuals who underwent WES and CMA and evaluated four WES-based CNV callers (CNVkit, CoNIFER, ExomeDepth, and cn.MOPS) against this benchmark. For each tool, we first defined software-specific problematic genomic regions across the full WES cohort and filtered out the CNVs that overlapped these regions. **Results**: The four algorithms showed low mutual concordance and distinct distributions in the problematic regions. On average, 2210 sequencing target baits (1.23%) were classified as problematic; these baits had lower mappability scores and higher coefficients of variation in RPKM than the remaining probes. After the supplementary filtration step, all tools demonstrated improved performance. Notably, ExomeDepth achieved gains of 14.4% in sensitivity and 7.9% in positive predictive value. **Conclusions**: We delineated software-specific problematic regions and demonstrated that targeted filtration markedly reduced false positives in WES-based CNV detection.

## 1. Introduction

A copy number variation (CNV) is a type of structural variation involving the gain or loss of a gene [1]. Many clinical studies of CNVs have revealed that these structural variations may be associated with disease phenotypes, including cancer, neurodegenerative disorders, immune deficiency, and multiple congenital anomaly syndromes [2,3,4]. Chromosomal microarray analysis (CMA) and multiplex ligation-dependent probe amplification (MLPA) are the gold standards for detecting CNVs in clinical settings. Both methods provide precise CNV identification and concordant results [5,6,7]. CNV detection using sequencing data has become an additional option with the advent of next-generation sequencing technologies. In the case of whole-genome sequencing (WGS) data, CNV and complex structural variations can be detected very accurately, but it is expensive and time-consuming [8,9]. In contrast, whole-exome sequencing (WES) is a cost- and time-effective method for detecting small variants and is also used to detect CNVs. Several programs have been developed for CNV detection using WGS and WES data, and most tools employ a read depth-based approach [10]. However, in the case of WES, detecting CNV accurately is complicated because of the variability in read depth that occurs during the experimental process or data analysis step. To solve these problems, each tool applies a unique statistical or analytical technique to normalize the read depth between samples and/or target regions. However, WES-based CNV analysis remains challenging because the results are inconsistent among different methods and have shown low sensitivity and high false positives in previous studies [11,12,13,14,15,16].

In this study, we estimated the performance of several CNV detection programs using a reference set from the CMA data and identified the characteristics of true-positive, false-negative, and false-positive CNV. In addition, we defined a problematic region for each program and applied an additional filtration step to reduce the false positives.

## 2. Materials and Methods

### 2.1. Sample Information

This retrospective quality-improvement study analyzed 180 unrelated clinical WES samples (86 females, 94 males; median age 12.4 years, range 0.2–68 years) from patients who underwent diagnostic testing at Korea University Anam Hospital and Guro Hospital between January 2021 and June 2023. Exome capture was performed using the Agilent SureSelect Human All Exon v6 kit (Agilent Technologies, Santa Clara, CA, USA), and libraries were sequenced on an Illumina NovaSeq 6000 (Illumina, San Diego, CA, USA) (2 × 150 bp, mean on-target depth 110×, ≥20× coverage for 97.8% of targets). Reads were aligned to GRCh37 using BWA-MEM (v0.7.17), duplicates were marked using Picard, and base-quality recalibration was performed using GATK (v4.2.2). This study was approved by the Institutional Review Board (IRB number: 2018GR0260, 2020AN0332). All participants or their legal guardians provided written informed consent, in accordance with the Declaration of Helsinki.

### 2.2. Identification of Copy Number Variations (CNVs) from Chromosomal Microarray Analysis (CMA)

The CMA data of 44 samples from 180 clinical WES samples were generated using Cytoscan DX and 750k chips. We used the chromosome analysis suite program (ChAS) to identify CNVs from the CMA data. The following filter conditions, which were applied in the previous study [17], were used to select high-quality, true positive CNVs from the CMA. CNVs that included fewer than ten SNP probes or were within highly polymorphic regions (present in more than four samples) were excluded. For comparison with sequencing platforms, we extracted CNVs that overlapped with at least one sequencing target bait. We then manually reviewed the log2Ratio and b-allele frequency of the CNVs and removed technical artifacts to ensure the highest quality of the CMA truth set. Among the 44 individuals with at least one CMA CNV, five did not contribute any events to the final truth set because none of their CNVs satisfied all of the above probe-count, polymorphic-region, bait-overlap, and manual quality filters. Consequently, the final reference set comprised 98 CNVs in 39 individuals, spanning 2788 exons, and all recall and PPV calculations in this study used these 39 CMA-positive individuals as the denominator.

### 2.3. CNV Identification from WES Data

WES data from 180 individuals were obtained using the SureSelect All Exon V6 Kit (Agilent Technologies, Santa Clara, CA, USA). Sequenced reads were aligned to GRCh37 using the Burrows-Wheeler Aligner-MEM algorithm [18], and duplicate reads were marked using Picard [19].

CNVs were detected using four programs: ExomeDepth (v1.1.15), CNVkit (v0.9.7), CoNIFER (v.0.2.2), and cn.MOPS (v1.14.1) [20,21,22,23]. ExomeDepth, CNVkit, and cn.MOPS were used with default parameters, whereas CONIFER was run with a singular value decomposition parameter of eight.

### 2.4. Performance Evaluation

To evaluate the accuracy of the programs, exons within the CNVs identified by CMA were considered as CNV exons and compared with the sequencing data. The following metrics were used to evaluate the performance of the CNV detection programs:

$$\mathrm{Sensitivity}=\frac{True\;Positive}{True\;Positive+False\;Negative},$$

$$\mathrm{Positive}\;\mathrm{predictive}\;\mathrm{value}\;(\mathrm{PPV})=\frac{True\;Positive}{True\;Positive+False\;Positve}$$

### 2.5. Defining Problematic Regions

To define problematic regions where CNVs were repeatedly called in multiple samples for each program, we merged the CNV-calling results and selected genomic regions where CNVs were identified in more than 10% of the cohort. The 35mer Duke uniqueness score [24] was used to calculate the mappability score for each target bait. The reads per kilobase million (RPKM) value for each target bait was obtained using CoNIFER and used to compute the intersample coefficient of variation (CV). The GC contents of the target exons were calculated using Bedtools [25]. To compare various genomic features between the problematic and non-problematic regions for each program, we randomly selected an equal number of probes in the non-problematic regions and used the Wilcoxon test to compare the two groups. We performed 1000 iterations of this analysis to test the effect of the random selection of probes.

### 2.6. Statistical Analysis

Comparisons of sensitivity and positive predictive value (PPV) before and after filtering were tested using a Wilcoxon signed-rank test; *p*-values < 0.05 were considered significant.

## 3. Results

### 3.1. Reference CNV Set Construction and Detection Program Evaluation

To evaluate the performance of the WES-based CNV detection programs, we constructed a reference set of CNVs using CMA data. The reference set of true-positive CNVs consisted of 98 CNVs, including 39 deletions and 59 duplications, in 39 samples (Table 1). In the WES-based analysis, we observed that the total number of CNVs called varied between programs. ExomeDepth had the highest number of CNVs, followed by cn.MOPS, CNVkit, and CoNIFER (10455, 1439, 782, and 544 CNVs, respectively). Most programs, except CoNIFER, tended to call more deletions than duplicates. The average size of the detected CNVs differed between the programs. ExomeDepth and cn.MOPS had shorter CNVs than the CNVkit or CoNIFER (Table 2). Of the 98 CNVs identified using CMA, 80 were also identified using WES. For CNVs smaller than 100 kb, no significant differences were observed between true positives and false negatives in terms of the mappability score or the coefficient of variation in reads-per-kilobase-per-million (RPKM CV). The only factor that showed a statistically significant difference between groups was the number of sequencing target baits used. For CNVs ranging between 100–500 kb, both the RPKM CV and the number of target baits exhibited statistically significant differences between true positives and false negatives (Table 3).

**Figure 1:**
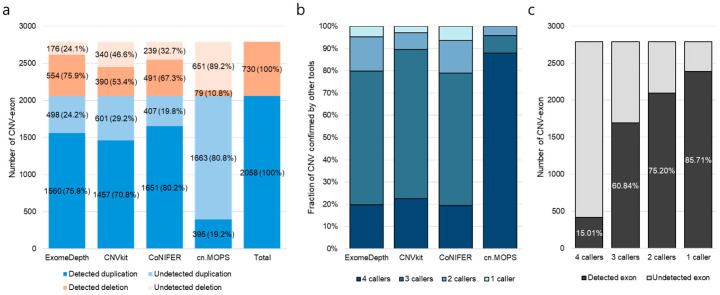
Detection of CNV-exons and tool concordance. (**a**) Number of CNV exons for the different CNV types. (**b**) Detection rate of CNVs using overlapping programs. (**c**) Fraction of CNV exons confirmed using other tools.

The CNVs from the CMA data included 2058 deleted exons and 730 duplicated exons. CoNIFER showed the highest sensitivity, followed by ExomeDepth, the CNV Kit, and cn.MOPS. (Figure 1a). Next, we checked the concordance between programs at the exon CNV level. Three programs, excluding cn.MOPS, were identified an average of 8% software-specific CNVs. Most CNVs identified in each program were detected by more than two callers, and all CNV exons detected by cn. MOPS were also detected by the other programs. (Figure 1b). Only 15% of the total true positives were found for all programs, and the combined results for all programs showed the highest sensitivity (85.71%) (Figure 1c). The mappability score and RPKM CV differed significantly between false and true positives. False-positive CNVs showed a low mean mappability score and high RPKM CV compared to true-positive CNVs, and this pattern was similarly identified in all programs (Supplementary Figure S1).

### 3.2. Problematic Region in CNV Detection

The problematic region was defined using WES data from 180 individuals and exhibited different distribution patterns across CNV detection programs. For example, the number of samples and probes within the CNV regions and the size of the detected regions differed between CNV detection programs in the killer cell immunoglobulin-like receptor region (Supplementary Figure S2).

**Figure 2:**
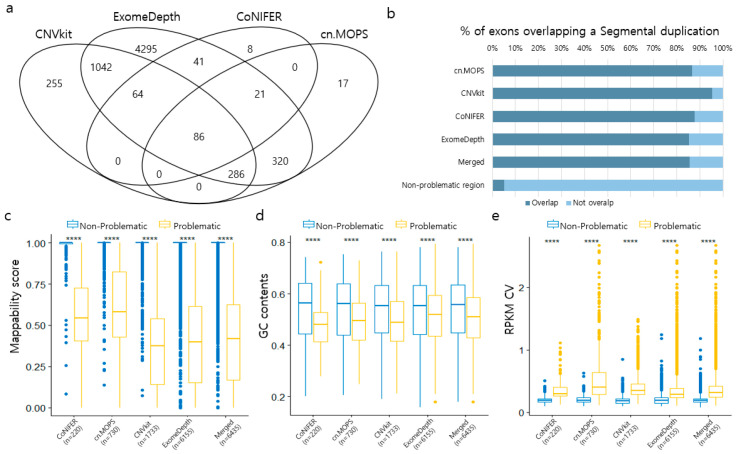
Characteristics of problematic regions. (**a**) Number of overlapping problematic exons between callers. (**b**) Percentage of exons overlapping with known Segmental Duplication (SD) regions, comparing non-problematic and problematic regions. (**c**) Mappability score, (**d**) GC content, and (**e**) RPKM CV of exons in the problematic region. Box plots comparing the distribution of key technical metrics between non-problematic (blue) and problematic (yellow) regions. Statistical Note: Asterisks * in panels (**c**–**e**) indicate the level of statistical significance (**** *p*-values < 0.0001) between the Problematic and Non-Problematic groups determined by the Wilcoxon test.

A total of 6435 probes were located in problematic regions across the four programs. Among these, 93% of the probes from the CNVkit, CoNIFER, and cn.MOPS overlapped with those from ExomeDepth. Notably, the majority of problematic probes (6155 of 6435) were observed in ExomeDepth, and only 220 probes were located in CoNIFER (Figure 2a). Among these probes, 2986, 987, 90, and 30 probes were included in the disease-associated genes, pseudogenes, HLA regions, and immunoglobulin-like receptor regions, respectively (Supplementary Table S2). More than 80% of the probes in problematic regions overlapped with segmental duplications, whereas only 5.2% of the probes in non-problematic regions overlapped with these duplications (Figure 2b).

Additionally, we found that sequencing baits in problematic regions had lower mappability scores and GC content than randomly selected probes in non-problematic regions. The RPKM CV was significantly high in the problematic regions (Figure 2c–e). This analysis was repeated 1000 times to estimate the reproducibility of the findings. We found that the distributions of mappability scores, GC content, and RPKM CVs of randomly selected probes in non-problematic regions were consistent across all programs for 1000 iterations. We also found that the mean of these features differed from those of the probes in the problematic regions (Supplementary Figure S3).

### 3.3. Performance Improvement

**Figure 3:**
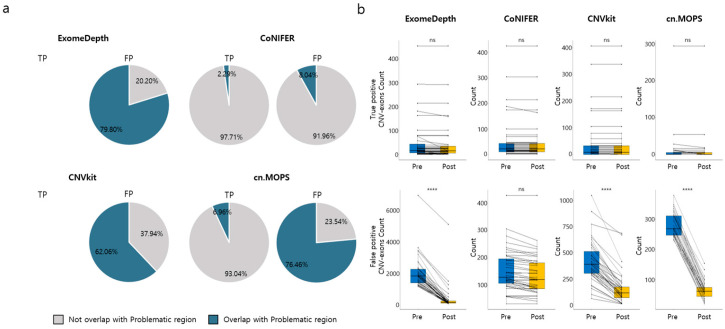
CNV filtration using information on problematic region (**a**) Fraction of the CNV-exons overlapped with problematic region for each program (**b**) The number of CNVs per individual before and after the filter. A connected line between the two spots indicates a change in the number of CNVs in the same sample. Statistical significance of changes between pre-filter and post-filter conditions was assessed using Wilcoxon signed-rank test. ns, not significant; **** *p* < 0.0001.

Analysis of false positives from the WES data suggested that a high proportion of CNVs overlapped with problematic regions. In particular, we found that an average of 73% of the false-positive CNVs from the three programs (ExomeDepth, CNVkit, and cn.MOPS) were located in these areas, whereas only 8% of the false positives from CoNIFER overlapped with problematic regions (Figure 3a). Information on problematic regions was used to filter CNVs from the WES data. By applying additional filtration, false-positive CNVs were significantly reduced, while the modest reduction in the number of true-positive CNVs was not statistically significant in ExomeDepth, CNVkit, and cn.MOPS. In the case of CoNIFER, although the false positives tended to decrease after filtering, the reduction was not statistically significant (Figure 3b). ExomeDepth and CoNIFER showed similar sensitivities, but the positive predictive value (PPV) of ExomeDepth was lower than that of CoNIFER under prefiltering conditions. After filtering the CNVs, we confirmed that the overall performance improved for all programs. In particular, the sensitivity of ExomeDepth increased from 0.758 to 0.903, and the PPV increased five-fold compared with that before filtration (Table 4).

## 4. Discussion

CNVs are important genomic variants that cause several genetic diseases. CMA and MLPA have been the gold standards for detecting CNVs in genetic diagnostics for more than a decade, and NGS techniques have provided alternative methods for CNV detection in the research field. Although various CNV detection programs have been developed, CNV analysis using WES data remains challenging [11]. In particular, the depth of coverage in some regions varies greatly among samples and probes because of technical artifacts caused by several features, such as different capture efficiencies, low sequence complexities, and high GC content. These regions result in many false-positives, making it difficult to detect true-positive CNVs.

In this study, we benchmarked WES-based CNV detection programs and provided information on the problematic regions for each program. In our CMA-positive cohort, the mean number of CNVs per individual was relatively low (1.95 events per case; Table 1). This likely reflects our stringent construction of the CMA truth set, which applied size and probe-count thresholds. However, some true CNVs may still have been missed by CMA, so the true CNV burden is probably higher than the number of events included in our reference set. Consistent with previous studies, we found that the CNV detection programs showed inconsistent results in terms of the number, size, and genomic regions of CNVs [11,26]. Among these, CoNIFER exhibited the best performance in terms of sensitivity and PPV. Although the sensitivity of ExomeDepth was similar to that of CoNIFER, ExomeDepth identified many false positives. As the detection results differed among the programs, the combined results of the four CNV detection programs showed the highest sensitivity. This finding is highly consistent with the recent literature, which confirms that ensemble approaches are critical for maximizing both the sensitivity and specificity of WES-based CNV detection in diagnostic settings [27,28,29].

A consensus regarding the need for ensemble calling has driven the development of new and sophisticated algorithms. Although our analysis was restricted to established callers, recent benchmarking studies have identified emerging tools such as ClinCNV and GATK-gCNV, which exhibit superior performance in terms of F1 score and sensitivity for germline CNV detection in gene panels and exome data [30,31]. Furthermore, novel ensemble methods, such as EMcnv, have shown favorable median precision (e.g., 0.93) compared to standard exome-optimized tools [28]. Our finding that CoNIFER performed optimally under the pre-filter condition was contextualized by the older tool set evaluated. Crucially, the methodology for identifying and filtering systematic noise via RPKM CV and mappability remains broadly applicable regardless of the core calling algorithm. This targeted approach aligns with previously suggested workflow modifications to exclude polymorphisms and false positives from repetitive regions in the ExomeDepth pipelines.

In previous studies using ExomeDepth for CNV detection, the mean mappability score of false-positive CNV was lower than that of true-positive [17]. We found that other software, as well as ExomeDepth, showed a similar pattern. The RPKM CV directly indicates inter- or intra-sample variability in depth for each exon and has a higher value in false-negative CNVs than in true-positive single-exon CNV analyses [32]. In this study, the RPKM CV tended to be slightly higher for false negatives; however, the difference was not statistically significant. We confirmed that the RPKM CV was significantly high in false positives.

Problematic regions can originate from repetitive regions of the genome with low mappability scores. Alignment of sequenced reads and downstream analyses has been inaccurately performed in these regions [33]. Highly polymorphic regions such as HLA and killer cell immunoglobulin-like receptor regions also have the potential to generate noise during CNV detection [34]. In this study, we operationally defined “problematic regions” for each caller as exons where a CNV was called in at least 10% of samples in our WES cohort. This recurrence-based criterion was chosen as a pragmatic, proof-of-principle threshold to capture exons that are disproportionately affected by systematic technical artifacts or benign polymorphic variation, rather than isolated, sample-specific events. We found that a high proportion of false-positive CNVs in WES overlapped with the problematic regions. Importantly, these recurrence-based regions were not used as a replacement for conventional genomic metrics such as mappability, GC content, or alignment depth, but rather as an additional, algorithm-adaptive mask applied on top of standard QC. This design acknowledges that different CNV callers exhibit distinct failure modes even within loci that appear acceptable by conventional metrics, and that recurrent artifacts can therefore reveal caller- and kit-specific problematic regions that are not fully captured by mappability- or GC-based filters alone. By filtering out CNVs in these regions, we selectively reduced the number of false positives, with only a modest and statistically non-significant reduction in true-positive CNV exons for ExomeDepth, CNVkit, and cn.MOPS. Using this filtration step, we were able to improve the overall performance of all the programs. In particular, the five-fold increase in the positive predictive value of ExomeDepth significantly enhanced clinical efficiency by reducing the required manual review and orthogonal validation of spurious calls.

Furthermore, we found that clinically significant genes were located in the problematic regions. Genes associated with developmental disorders and hereditary cancer syndromes were identified in these regions, emphasizing the importance of accurate CNV detection. Notably, 23 of the 404 genes harbored ACMG Tier genes (e.g., *PMS2* and *SMN1*). CNV detection in genes such as *PMS2* (Lynch syndrome) and *SMN1* (Spinal Muscular Atrophy) is notoriously challenging because of high-similarity paralogs (*PMS2CL* and *SMN2*) that cause nonspecific read alignment and distorted read depth [35]. Therefore, we recommend orthogonal confirmation (MLPA or CMA) whenever a candidate CNV intersects with these loci [36,37]. This dual approach ensures the integrity and safety of clinical molecular diagnostics, particularly in regions inherently prone to technical artifacts.

This study had several limitations. First, we defined the problematic regions only for the SureSelect WES V6 Target Capture Kit, using a single recurrence threshold of 10% to flag exons with systematic noise. Because both the target sequencing design and cohort characteristics differ across kits and laboratories, this numerical cut-off should be regarded as a cohort- and kit-specific, proof-of-principle choice rather than a universal standard. In other settings, it will be necessary to empirically explore alternative recurrence thresholds (e.g., 5–15%) and select the value that provides the most favorable balance between sensitivity and positive predictive value. Therefore, testing other kits and tuning the recurrence cut-off in a data-driven manner is necessary. The second limitation is that filtering of the problematic region excludes probes in some disease-associated genes. The sequencing target baits of 404 clinically relevant genes were included in these problematic regions. For accurate detection, the CNVs in these genes require further validation using other orthogonal methods. Finally, a third limitation concerns the version of the human reference genome used, as this study relied on the older GRCh37 (hg19) reference sequence. GRCh38 (hg38) is the more recent and accurate version, featuring critical improvements such as corrections for assembly errors, a reduction in genomic gap regions, and the inclusion of additional alternate loci. The use of GRCh37 may have slightly constrained the accuracy of read alignment and downstream variant calling.

Future studies should validate the problematic region catalogue in an independent whole-exome cohort that employs a distinct capture design. The application of the current filter to such data, together with systematic evaluation of alternative recurrence thresholds, would permit an unbiased estimate of its effect on sensitivity and positive predictive value and help define cohort-specific cut-offs. In addition, assessing how recurrence-based problematic regions interact with mappability-, GC-, and depth-based filters in other pipelines will be important for demonstrating the generalizability of this combined, two-layer strategy across laboratories.

In conclusion, this study provides a rigorous framework for assessing and improving the performance of WES-based CNV detection. By systematically identifying and filtering regions defined by recurrent, algorithm-specific technical noise in conjunction with conventional genomic metrics, we demonstrated a dramatic enhancement in caller specificity, bringing WES closer to becoming a robust single-platform solution for comprehensive molecular diagnostics.

## Figures and Tables

**Table 1:** Construction of a reference CNV set from chromosomal microarray data.

	High Quality True-Positive CNV	
	Deletions	Duplications
Number of CNVs	39	59
Size of CNVs	5.03 kb–4.04 Mb (mean = 292.42 kb)	53.47 kb–6.91 Mb (mean = 594.91 kb)
Number of CNVs per individual	1–4 (mean = 1.95)	1–6 (mean = 1.79)

**Table 2:** Results of CNV analysis using WES data with default parameters.

*N* = 39	ExomeDepth		CNVkit		CoNIFER		cn.MOPS	
	Del	Dup	Del	Dup	Del	Dup	Del	Dup
Total call	5733	4722	430	352	198	346	951	488
Size of CNVs	0.32 kb–3.98 Mb (33.15 kb)	0.32 kb–5.14 Mb (49.31 kb)	0.40 kb–3.98 Mb (143.56 kb)	3.25 kb–6.95 Mb (279.49 kb)	2.90 kb–4.88 Mb (178.81 kb)	2.09 kb–21.54 Mb (277.20 kb)	2.99 kb–3.59 Mb (41.63 kb)	3.37 kb–2.96 Mb (49.56 kb)
Number of CNVs per individual	85–233 (147.0)	58–228 (121.08)	3–23 (11.03)	1–23 (9.03)	0–14 (5.08)	2–25 (8.87)	18–30 (24.38)	7–24 (12.51)

**Table 3:** Characteristics of true-positive and false-negative CNVs. Asterisk denotes a statistically significant difference between true positives and false negatives.

Size of CNVs		True Positive	False Negative
−100 kb	Number of CNVs	31	9
	Mappability score	0.41–1 (mean = 0.88, median = 0.98)	0.44–1 (mean = 0.92, median = 1)
	RPKM CV	0.16–0.57 (mean = 0.23, median = 0.19)	0.17–0.57 (mean = 0.27, median = 0.23)
	Number of WES target baits *	1–52 (mean = 9.13, median = 5)	1–3 (mean = 1.4, median = 1)
100 kb–500 kb	Number of CNVs	32	7
	Mappability score	0.24–1 (mean = 0.77, median = 0.86)	0.24–0.92 (mean = 0.69, median = 0.82)
	RPKM CV *	0.19–0.46 (mean = 0.26, median = 0.23)	0.24–0.59 (mean = 0.44, median = 0.47)
	Number of WES target baits *	1–104 (mean = 29.28, median = 21)	1–72 (mean = 13.57, median = 3)
500 kb	Number of CNVs	17	2
	Mappability score	0.28–1 (mean = 0.84, median = 0.96)	0.61–1 (mean = 0.81, median = 0.81)
	RPKM CV	0.14–0.27 (mean = 0.21, median = 0.21)	0.19–0.31 (mean = 0.25, median = 0.25)
	Number of WES target baits	2–308 (mean = 85.23, median = 66)	1–10 (mean = 5.5, median = 5.5)

* indicates statistically significant difference between true positives and false negatives (*p* < 0.05).

**Table 4:** Performance of the four CNV detection programs.

	ExomeDepth		CNVkit		CoNIFER		cn.MOPS	
	Pre-filter	Post-filter	Pre-filter	Post-filter	Pre-filter	Post-filter	Pre-filter	Post-filter
Reference Truth set	2788	2124	2788	2710	2788	2713	2788	2723
TP	2114	1917	1847	1844	2142	2093	474	441
FP	83,135	16,797	17,340	6578	6838	6288	11,164	2628
FN	674	207	941	866	646	620	2314	2282
Sensitivity	0.758	0.903	0.662	0.680	0.768	0.771	0.170	0.162
PPV	0.025	0.102	0.096	0.219	0.239	0.250	0.041	0.144

## Data Availability

The data supporting the findings of this study are included within the article and its Supplementary Materials. Additional details on genomic regions, probe characteristics, and CNV-related datasets generated and analyzed during this study are available in the supplementary files provided with the manuscript. Further inquiries can be directed to the corresponding author upon reasonable request.

## Supplementary Materials

The following supporting information can be downloaded at: https://www.mdpi.com/article/10.3390/genes17010105/s1, Supplementary Figure S1. Difference in mappability score and RPKM CV between true positives and false positives for each program; Supplementary Figure S2. Genome-wide distribution and density of problematically recurrent CNV regions. This figure illustrates the distribution and relative density of the regions identified as being recurrently affected by CNV calls across all human chromosomes, as identified by each of the four CNV calling tools. The variation in coverage and algorithmic strategy across tools results in distinct sets of problematic regions. Colored Lines/Bars: Represent the genomic density (or frequency) of problematic CNV calls for each specific caller (e.g., Red for Tool A, Blue for Tool B, etc.). Red Box: Highlights a known example of a Highly Polymorphic (HP) genomic region, such as the Killer cell immunoglobulin-like receptor (KIR) gene region in chromosome 19. This area is characterized by complex duplications, making it prone to high sequencing noise and unreliable CNV calling. Supplementary Figure S3. Distribution of average mappability score, GC content, and RPKM CV. For each program, probes were randomly selected in the non-problematic region and the average of each feature was calculated for 1000 iterations. The dotted lines indicate the mean values for each program; Supplementary Table S1. Genomic coordinates of problematic region; Supplementary Table S2. Detailed information on 404 genes in the problematic region (BED + HUGO symbol + OMIM phenotype).

## Abbreviations

The following abbreviations are used in this manuscript:

CI	confidence intervals
CMA	chromosomal microarray analysis
CNV	copy number variation
CV	coefficient of variation
MLPA	multiplex ligation-dependent probe amplification
PPV	Positive predictive value
RPKM	reads per kilobase million
WES	Whole exome sequencing
WGS	whole-genome sequencing
