# Supplementary figures and images for: Improving CNV Detection Performance Except for Software-Specific Problematic Regions
Source: Genes (Basel). 2026 Jan 19;17(1):105. doi: 10.3390/genes17010105 (PMC12841491; doi:10.3390/genes17010105)

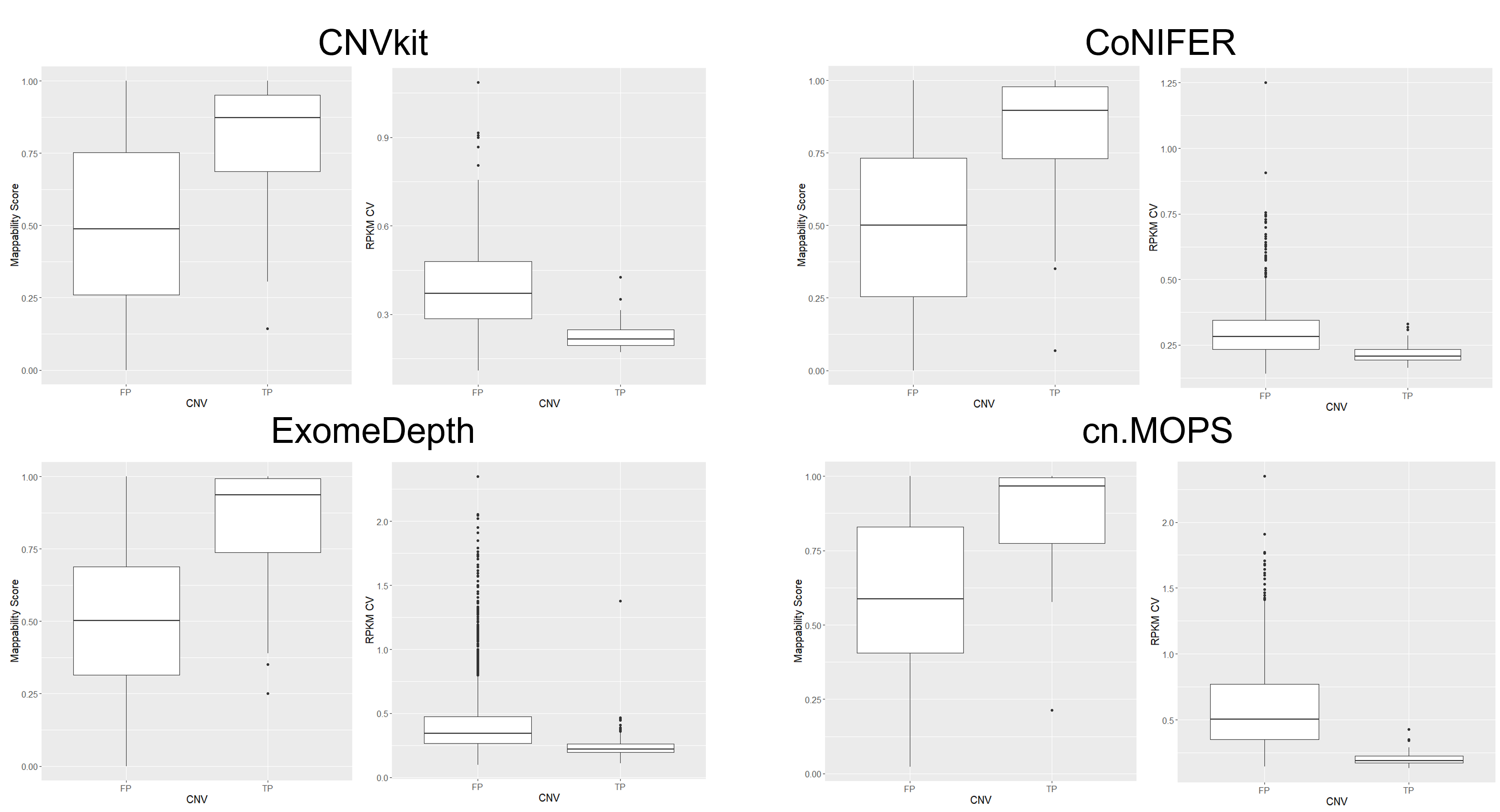

Supplement: Supplementary file 1 [file genes-17-00105-s001.zip › figureS1.png]

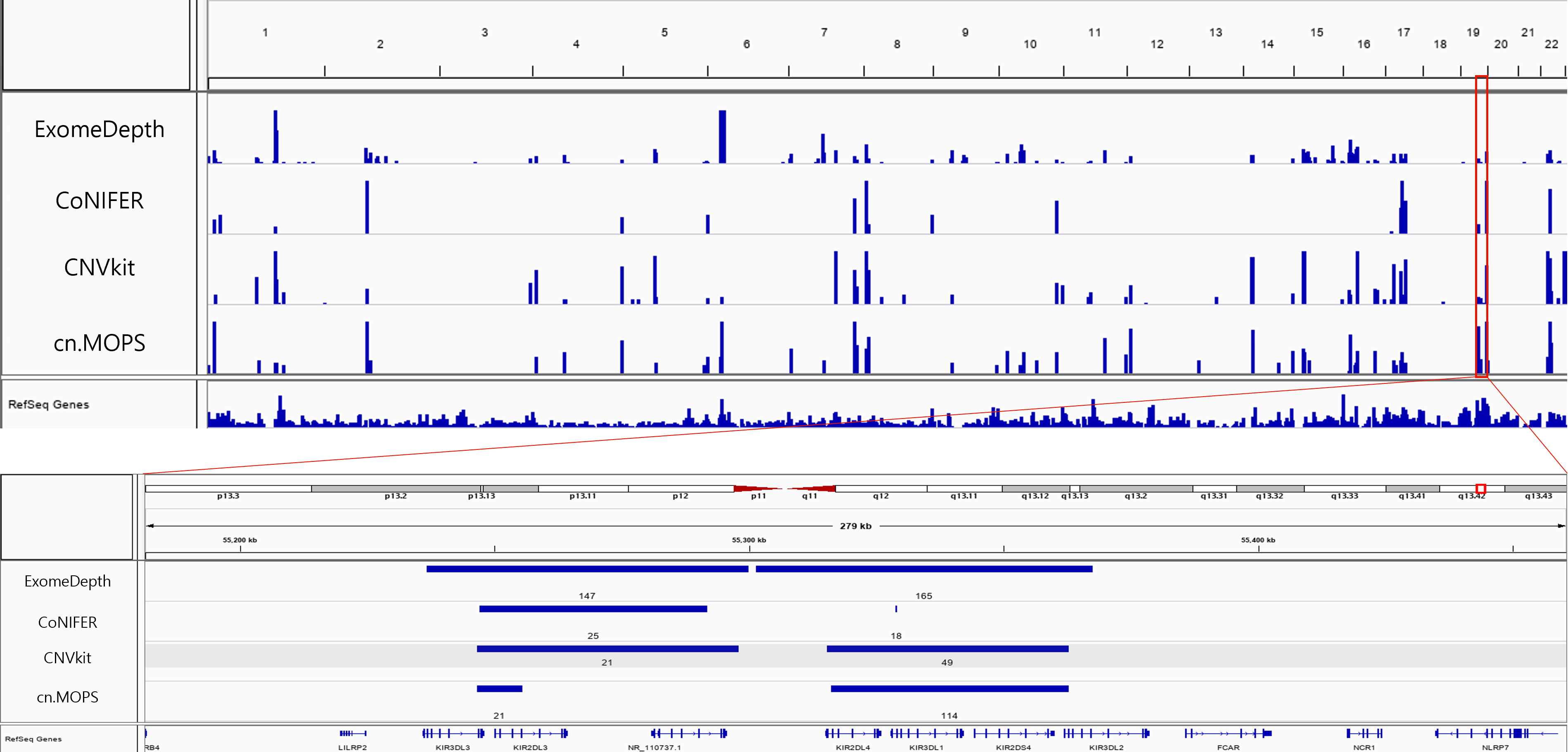

Supplement: Supplementary file 1 [file genes-17-00105-s001.zip › figureS2.png]

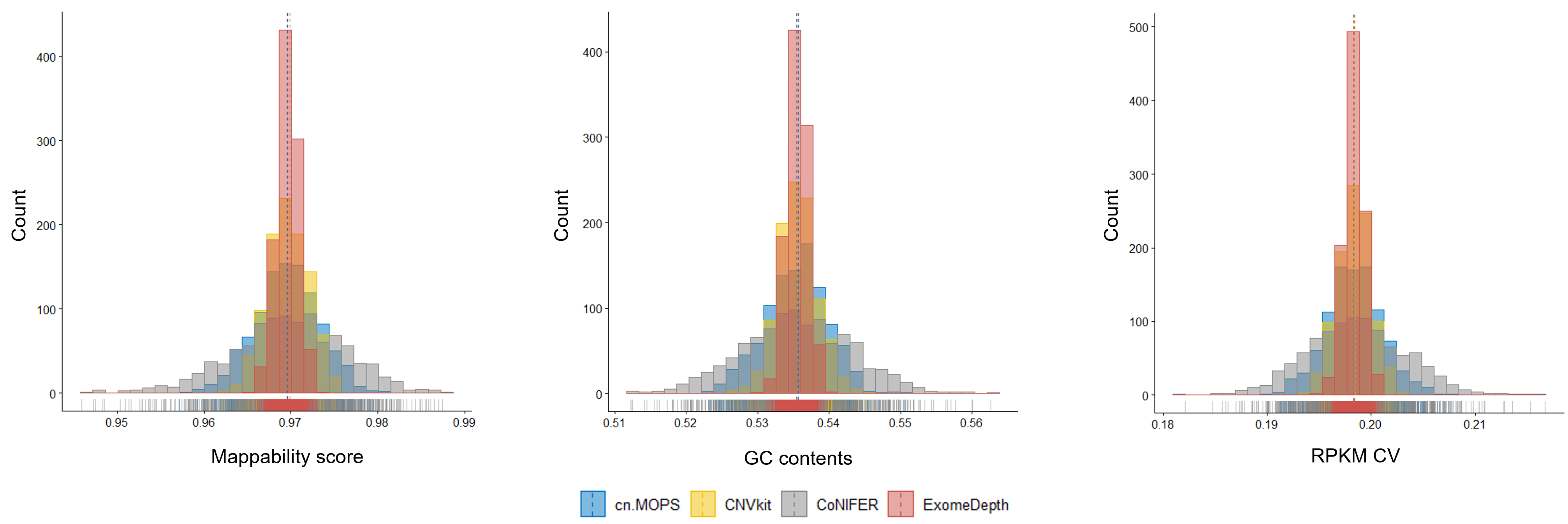

Supplement: Supplementary file 1 [file genes-17-00105-s001.zip › figureS3.png]
